# Endovascular reperfusion followed by delayed open aortic repair in stable acute type A aortic dissection with malperfusion syndrome: a single-center experience

**DOI:** 10.3389/fmed.2025.1701176

**Published:** 2026-02-11

**Authors:** Mengmeng Ye, Chao Xue, Dongxu Wang, Yi Si, Yong Mao, Bo Xu, Bo Yu, Weiguang Wang, Kai Ren, Jian Xu, Hongliang Zhao, Shiqiang Yu, Jincheng Liu, Weixun Duan

**Affiliations:** 1 Department of Cardiovascular Surgery, Xijing Hospital, The Air Force Medical University, Xi'an, Shaanxi, China; 2 Department of Cardiac Surgery, The Second Hospital and Clinical Medical School, Lanzhou University, Lanzhou, Gansu, China

**Keywords:** acute aortic dissection, type A, malperfusion syndrome, organ failure, reperfusion

## Abstract

**Background:**

We aimed to evaluate the outcomes of endovascular reperfusion followed by delayed open aortic repair in patients with acute type A aortic dissection (ATAAD) and malperfusion syndrome (MPS), as well as risk factors for mortality associated with organ failure.

**Methods:**

We retrospectively selected 777 patients with ATAAD admitted to our center. Patients with MPS (*n* = 121; 15.6%), who were hemodynamically stable and without evidence of aortic rupture/tamponade, underwent interventional reperfusion (IR) through aortic/mesenteric branch stenting, followed by delayed open aortic repair (OR). Patients without MPS (non-MPS) (*n* = 656; 84.4%) received immediate open aortic repair.

**Results:**

Overall hospital mortality was 37.2% in patients with ATAAD and MPS, significantly higher than in those without MPS (8.2%). However, patients with MPS who successfully underwent delayed repair after stenting had hospital mortality rates comparable to non-MPS patients (11.7% vs. 8.2%; *p* = 0.306) and similar short-term survival. Hypertension, leukocytosis, fibrin degradation products (FDP), D-dimer, and FDP/D-dimer ratios were independent predictors of mortality associated with irreversible organ failure.

**Conclusion:**

Endovascular stenting followed by delayed open aortic repair in stable patients with ATAAD and MPS had favorable short-term outcomes. Stenting of the aorta and/or aortic branches is a relatively simple, minimally invasive intervention with short-term patency.

## Introduction

Acute type A aortic dissection (ATAAD) is a life-threatening cardiovascular disease with high short-term morbidity and mortality. The mortality rate increases by 1–2% per hour in the first 48 h, resulting in a mortality rate of approximately 50% without timely surgical intervention (1).

Despite immediate surgical repair, the perioperative mortality in patients with ATAAD ranges between 21 and 42%. Patients presenting with malperfusion involving more than one end-organ system have a mortality rate of 86% (2–5). Malperfusion occurs in up to 44% of patients with ATAAD, and despite improved surgical techniques, additional vascular intervention is required in one-third of patients with malperfusion syndrome (MPS) (4). According to a study conducted by the International Registry of Acute Aortic Dissection, increased hospital mortality in ATAAD patients is associated with mesenteric malperfusion, coma, limb ischemia, and coronary malperfusion (6).

The definitive treatment for ATAAD remains open surgical repair, which aims to respect the primary entry tear and prevent aortic rupture. However, in view of the high hospital mortality rate, Deeb et al. (7) proposed interventional radiology (IR) followed by delayed open aortic repair (OR) for stable patients with ATAAD and MPS and achieved a significant reduction in mortality (from 89 to 25%). Some centers have adopted this strategy to improve organ malperfusion and have reported that the mortality rate in patients with MPS is comparable to that in patients without MPS (3, 8–11). Nevertheless, the current literature on delayed open repair in patients with ATAAD and MPS remains limited to studies with small patient cohorts.

Given the high mortality associated with MPS in ATAAD patients and the limited evidence supporting delayed open repair strategies, we evaluated the effectiveness of delayed open aortic repair in a large cohort and identified the risk factors for mortality associated with organ failure.

## Methods

### Patient population and data collection

**Figure 1:**
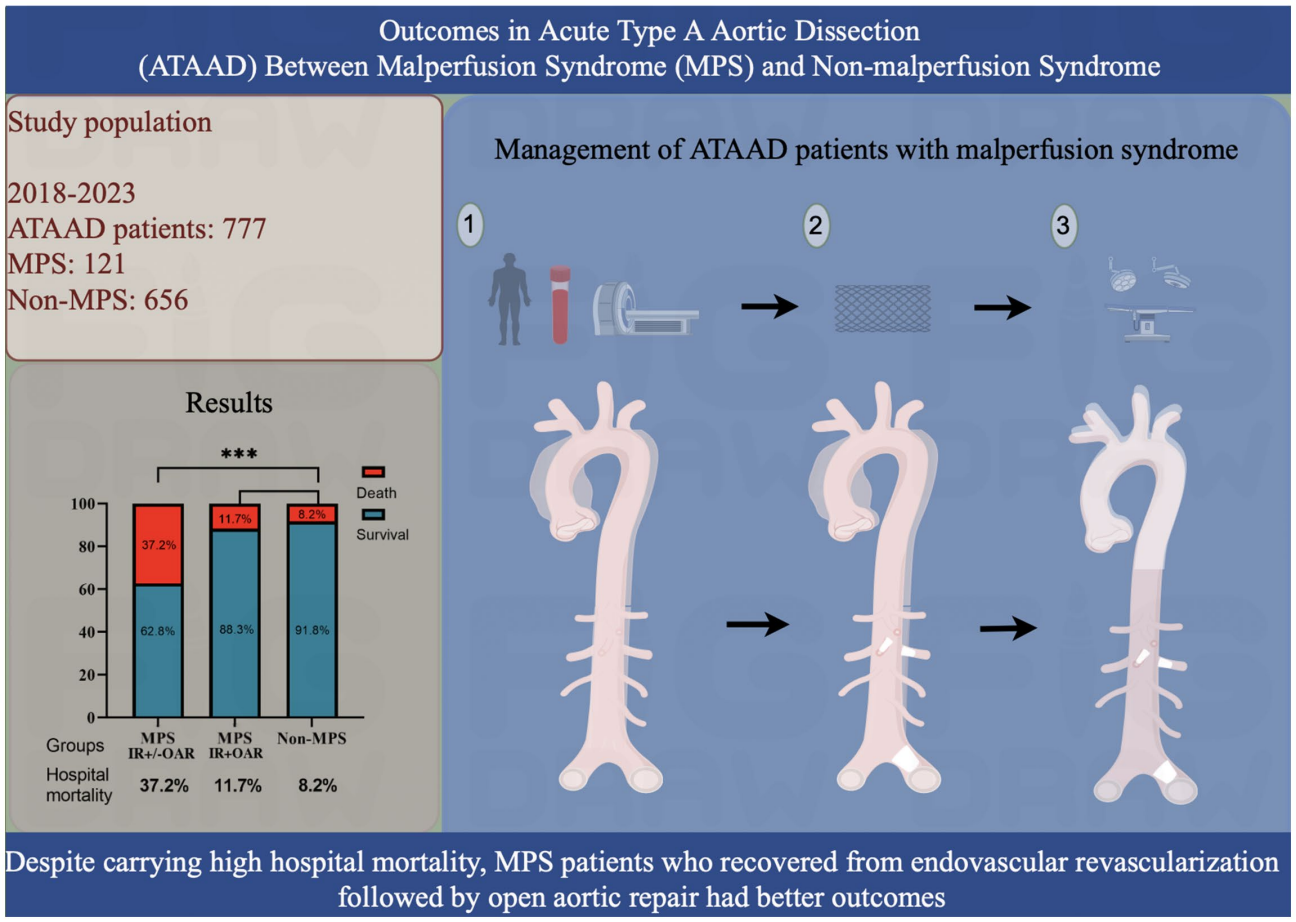
Outcomes in acute type A aortic dissection between patients in the malperfusion syndrome (MPS) and those in the non-MPS groups.

This single-center retrospective cohort study included 777 selected patients who had DeBakey type I dissection and were presented with ATAAD (defined as the onset of symptoms within 14 days of admission) between January 2018 and March 2022 (Figure 1). The enrolled patients were divided into two groups: the MPS group (*n* = 121; 15.6%) and the non-MPS group (*n* = 656; 84.4%). MPS patients with hemodynamic instability (e.g., evidence of aortic rupture/tamponade, severe aortic regurgitation with compromise, or refractory shock) underwent emergent open repair and were thus excluded from this analysis. This retrospective study complied with the Declaration of Helsinki (2000) and was approved by the Institutional Review Board of Xijing Hospital affiliated with the Fourth Military Medical University (20120216-4). Written informed consent was obtained from all patients to include their information in this publication.

### Diagnosis and management of ATAAD MPS

Malperfusion refers to compromised blood flow to an organ or limb caused by aortic dissection, confirmed by imaging. MPS is defined as late-stage malperfusion leading to tissue or organ damage (necrosis and/or end-organ dysfunction). Diagnosis requires both clinical features, laboratory findings, and radiographic evidence of malperfusion.

The types of MPS included coronary MPS (changes in the electrocardiogram with elevated troponin, coronary trunk lesions on CTA), cerebral MPS (new-onset focal or generalized deficit with CTA or magnetic resonance imaging), mesenteric MPS (abdominal pain, bloody diarrhea, increased serum lactate, and inadequate contrast enhancement of the gut), renal MPS (acute kidney injury per KDIGO criteria: serum creatinine ≥0.3 mg/dL within 48 h or ≥1.5-fold increase from baseline, CTA evidence of malperfusion in ≥1 renal artery segment), and extremity MPS (motor/sensory dysfunction, absence of pulses, elevated myoglobin or creatine kinase, compromised flow to the lower extremity).

All patients with MPS are hemodynamically stable and preferentially treated with IR before delayed surgical repair. Extremity and mesenteric MPS were prioritized for intervention because of their higher morbidity and mortality, whereas renal MPS was managed conservatively owing to its compensatory capacity during cardiopulmonary bypass. For cerebral MPS, the primary goal is immediate revascularization. The approach was individualized based on anatomy and clinical presentation. Extra-anatomical revascularization was preferentially used for rapid restoration of cerebral flow, particularly in patients without profound coma (Supplementary Data). Endovascular stenting was considered in patients with amenable anatomy, especially in comatose patients where a minimally invasive approach was initially attempted.

Following interventional treatment, the decision to proceed to delayed open repair was based on observed trends in organ function recovery (Supplementary Video 1). Recovery was defined as stabilization or a reduction in vasopressor requirements, improvement in metabolic acidosis, resolution of abdominal pain and tenderness in patients with mesenteric MPS, return of palpable pulses and improvement in capillary refill in patients with limb MPS, and stabilization or improvement in neurological status in patients with cerebral MPS. Aortic repair was typically undertaken once initial stabilization was achieved, with a median target time from IR to OR of 0.6 days (IQR 0.4–0.9). The competing risk of aortic rupture was managed through intensive ICU monitoring. The basic management included adequate sedation, analgesia, and blood pressure control. Additionally, diuresis, urine alkalinization, intracranial pressure reduction, dialysis, and antimicrobial therapy were provided, depending on organ involvement. Perioperative anticoagulation with heparin was administered to all patients, and single antiplatelet therapy (aspirin) was required after surgery in patients without valve replacement.

## Interventional radiology procedures

The primary reperfusion strategy included endovascular stenting. Fenestration was not routinely performed; however, in cases with persistent obstruction or sluggish flow after stenting, selective thrombolysis was employed as an adjunct.

### Aortic bare-metal stenting

To address the problem of distal arterial obstruction resulting from a dissection flap prolapsing across the orifice of the branch vessels, distal aortic bare-metal stent grafts were performed percutaneously by covering or reaching the branches (Epic, 10–80 mm; Boston Scientific, Marlborough, MA, USA).

### Stenting of the aortic branch

Branch artery dissection with false lumen thrombosis (static type) was confirmed by selective arteriography, and stents were implanted into the true lumen, distally past the terminal extent of the dissection. The stents included Epic (8–60 mm and 10–100 mm; Boston Scientific), Innova (8–60 mm, 7–60 mm, and 6–40 mm; Boston Scientific), and JWS coronary balloon-coated stents.

### Aortic stenting plus stenting of branches

When the aortic dissection flap extends into the branches, which results in thrombosis of the false lumen and compression of the true lumen, combined aortic and branch stenting was performed to improve the end-organ blood supply.

### Thrombolytic catheter implantation

In the absence of distal arterial visualization or slow blood flow after stenting, as shown by angiography, a selective thrombolytic catheter was employed to resolve the thrombus in the true lumen of the branches (Cook Medical, Bloomington, IN, USA).

### Statistical analysis

This study was a retrospective analysis of a prospectively maintained database. Statistical analyses were performed using R software (Version 4.4.0; R Core Team, Vienna, Austria). Differences in numeric parameters between the two groups were assessed using the Student’s *t*- or the Mann–Whitney test. The Wilcoxon rank-sum test was applied for non-parametric comparisons of continuous variables. The chi-square or Fisher’s exact test was used to analyze categorical data. Variables in the univariate analysis (*p* < 0.1) were incorporated into a stepwise multifactor logistic regression analysis; variables at *p < 0.05 were considered statistically significant. Survival was analyzed using the Kaplan–Meier analysis. The graph abstract was drawn using Figdraw.

## Results

### Demographics of patients with and without MPS

A total of 777 patients were included in the study (mean age, 50 years; 78.5% males), and 15.6% (*n* = 121) had MPS. Patients with MPS exhibited poorer health indices than those without MPS, including a higher BMI (26.8 vs. 24.5, *p* < 0.001), a greater prevalence of previous cerebral infarction (5% vs. 0.9%, *p* < 0.001), and an increased rate of chronic kidney disease (2.5% vs. 0.6%, *p* = 0.032) (Table 1).

**Table 1:** Demographics and preoperative characteristics of all patients.

Admission variable	All patients (*n* = 777)	MPS (*n* = 121)	Non-MPS (*n* = 656)	*p*-value
Age, years	50 ± 10.6	49 ± 10.3	50.6 ± 10.4	0.134
Sex, male	612 (78.5)	105 (86.8)	507 (77.3)	0.061
BMI	24.9 (22.5, 27)	26.8 (24.7, 31.2)	24.5 (22, 26.3)	* < 0.001
Hypertension	471 (60.6)	70 (57.9)	401 (61.1)	0.516
Diabetes	13 (1.7)	0 (0)	13 (2)	0.118
Smoking	407 (52.4)	61 (50.4)	346 (52.7)	0.637
Past history				
Previous cardiac surgery	24 (3.1)	5 (4.1)	19 (2.9)	0.368
History of coronary artery disease	16 (2.3)	3 (2.5)	13 (2)	0.623
History of cerebral infarction	12 (1.5)	6 (5.0)	6 (0.9)	* < 0.001
History chronic kidney disease	7 (0.9)	3 (2.5)	4 (0.6)	*0.032
History of respiratory disease	25 (3.2)	5 (4.13)	20 (3)	0.424
Type of MPS				
Cerebral MPS	NA	14 (11.6)	NA	
Coronary MPS	NA	1 (0.8)	NA	
Celiac/hepatic MPS	NA	4 (3.3)	NA	
Mesenteric MPS	NA	37 (30.6)	NA	
Renal MPS	NA	32 (26.4)	NA	
Extremity MPS	NA	79 (65.3)	NA	
Aortic insufficiency	173 (22.3)	28 (23.1)	145 (22.1)	0.801
Time from symptom onset to admission, days	NA	0.3 (0.2, 0.5)	NA	
Time from symptom onset to IR, days	NA	0.6 (0.4, 0.8)	NA	
Time from IR to OR, days	NA	0.6 (0.4, 0.9)	NA	
Time from symptom onset to OR, days	1 (0.1, 1.1)	1.2 (0.9, 1.7)	0.8 (0, 1.0)	0.295

MPS, malperfusion syndrome; Non-MPS, no malperfusion syndrome; BMI, body mass index; IR, interventional radiology; OR, open aortic repair. *p < 0.05 was considered to be significantly different.

### Overall outcomes of patients with ATAAD and MPS

Extremity MPS (65.3%, 79/121) and mesenteric MPS (30.6%, 37/121) were the most common types requiring priority treatment (Table 1). None of these patients required bowel resection. Two patients underwent fasciotomy and dialysis due to compartment syndrome and hypermyoglobinemia. Among the 14 patients with cerebral MPS who achieved technical success and profound neurological recovery, 12 without coma were treated with extra-anatomical revascularization before aortic repair, while 2 with coma were attempted with endovascular stenting (Table 2). One hemodynamically stable patient with coronary MPS underwent bridging stenting. Only 5 of 32 patients with renal MPS received endovascular stenting (Table 2).

**Table 2:** Type of interventional radiology-amenable malperfusion syndrome.

	Stent in branch	Stent in aorta	Stent in branch + aorta	Stent in branch + aorta + contact thrombolysis	Percent dead per group, *n* (%)
Cerebral MPS	2	_	_	_	1 (0.8)
Coronary MPS	1	_	_	_	0
Celiac/hepatic MPS	_	_	_	_	2 (1.7)
Mesenteric MPS	27	8	2	_	16 (13.2)
Renal MPS	5	_	_	_	3 (2.5)
Extremity MPS	74	_	3	2	25 (20.7)

MPS, malperfusion syndrome.

**Figure 2:**
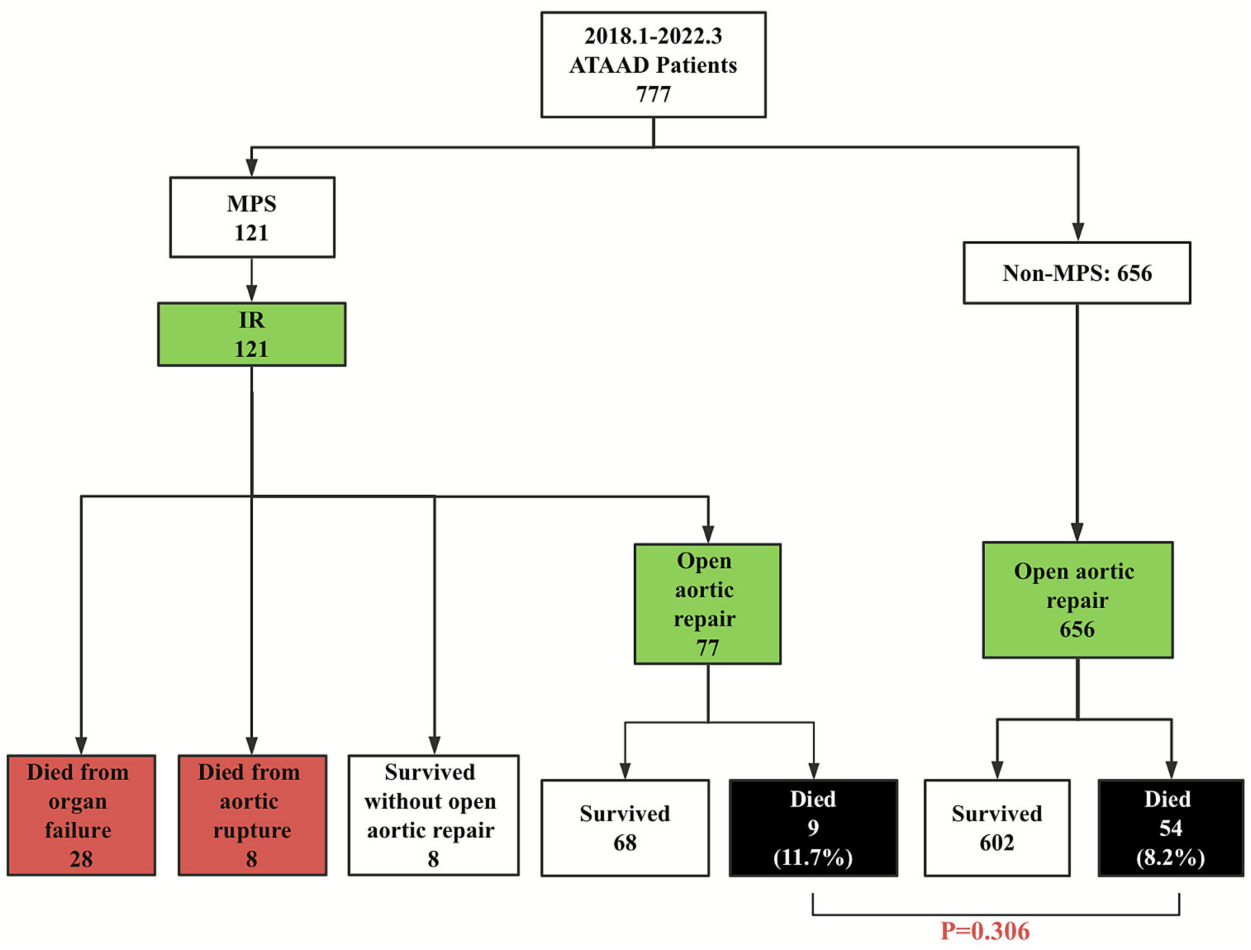
Management and short-term outcomes of patients with malperfusion syndrome. IR, interventional radiology; MPS, malperfusion syndrome.

Among 121 patients with MPS treated by percutaneous IR, 29.8% (36/121) patients died before OR (organ failure, *n* = 28; aortic rupture, *n* = 8) and 6.6% (8/121) survived through discharge without OR (the patients’ families declined further high-risk surgical intervention due to the patient’s critical condition). The remaining 63.6% (77/121) successfully underwent delayed OR (Figure 2; Table 3). The time from symptom onset to admission was 0.3 days (interquartile range 0.2–0.5) in patients with MPS, and the time from IR to OR was 0.6 days (IQR 0.4–0.9). There were no significant differences in the time from symptom onset to OR (1.2 vs. 0.8 days, IQR 0.9–1.7, *p* = 0.295) between patients in the MPS group and those in the non-MPS group (Table 1).

**Table 3:** Clinical condition of patients with malperfusion syndrome divided into three groups based on the outcome of endovascular reperfusion.

Admission variable	Death from aortic rupture (*n* = 8)	Survival† (*n* = 85)	Death from organ failure (*n* = 28)	*p*-valueǂ
Age, years	50.8 ± 9.1	47.5 ± 10.6	52.1 ± 8.9	*0.043
Gender, male	7 (87.5)	73 (85.9)	25 (89.3)	0.645
BMI	27 (21.7, 30)	25 (22.5, 26.9)	25 (22.5, 27.8)	0.267
Hypertension	3 (37.5)	53 (62.4)	12 (42.9)	*0.001
Smoking	3 (37.5)	47 (56.5)	11 (39.3)	0.142
Past history				
Respiratory disease	2 (25)	2 (2.4)	1 (3.6)	0.728
Coronary artery disease	0 (0)	2 (2.4)	1 (3.6)	0.728
Previous cardiac surgery	0	5 (5.9)	0	0.189
Cerebral accident	1 (12.5)	3 (3.5)	2 (7.1)	0.446
Chronic kidney disease	1 (12.5)	0	2 (7.1)	*0.013
Aortic insufficiency	3 (37.5)	21 (24.7)	4 (14.3)	0.249
Pre-AKI	2 (25)	2 (2.4)	13 (46.4)	* < 0.001
Laboratory examination				
WBC (10^9^/L)	15.2 (12.9, 16.8)	14.1 (11.4, 17)	17.1 (13.2, 19.2)	*0.031
NEU (10^9^/L)	13.1 (10.4, 14.5)	11.6 (9.4, 14.9)	14.6 (10.8, 17.2)	0.067
LYM (10^9^/L)	1.2 (0.7, 1.6)	1.0 (0.6, 1.5)	0.9 (0.5, 1.4)	0.587
RBC (10^12^/L)	4.3 (4.1, 4.4)	4.5 (4.1, 4.9)	4.5 (4.2, 5.0)	0.614
PLT (10^9^/L)	131 (103.5, 136.8)	158 (135, 199)	173 (126.8, 202)	0.676
NLR	12.7 (8.1, 20.8)	11 (7.5, 14.9)	19 (10.3, 25.7)	0.059
PLR	9.8 (7, 12.4)	17.6 (10.2, 20.3)	8.7 (5.7, 16.1)	0.127
PT (s)	16.1 (12, 22.1)	11.9 (11, 15.1)	12.6 (11.4, 19.3)	0.059
APTT (s)	32.9 (27.1, 37.8)	27.8 (24.7, 33.4)	28 (26.1, 31.7)	0.542
FDP (μg/mL)	70.9 (33.8, 134)	36.1 (22.8, 78.7)	104.5 (51.1, 188.7)	* < 0.001
D-dimer (μg/mL)	21.9 (7.3, 44)	9.2 (4.8, 20.6)	33.3 (14.5, 60.4)	* < 0.001
Ratio of FDP/D-dimer	4.1 (3, 4.6)	3.6 (3.1, 4.5)	3.2 (2.7, 4)	*0.040
Lac (mmol/L)	2.8 (2.3, 3.3)	2.7 (1.6, 4.2)	3.7 (3, 6)	*0.013
Creatinine (μmol/L)	115 (89.3, 131.8)	89 (73, 113)	115.5 (101.5, 201.8)	* < 0.001
cTnI (μg/L)	0 (0, 0.1)	0 (0, 0.4)	0.1 (0, 0.4)	0.677
Myoglobin (ng/mL)	261 (126, 570)	152 (51, 998)	668 (157, 1992)	*0.023

BMI, body mass index; Pre-AKI, preoperative acute kidney injury; WBC, white blood cell; NEU, neutrophil; LYM, lymphocyte; RBC, red blood cell; PLT, platelet; NLR, neutrophil-lymphocyte ratio; PLR, platelet-lymphocyte ratio; PT, prothrombin time; APTT, activated partial thromboplastin time; FDP, fibrin degradation product; Lac, lactate. *p < 0.05 were considered to be significantly different; †, defined as survival to open aortic repair (OR) or discharge without OR; ‡, the difference between the groups of death from organ failure and survival to open aortic repair or hospital discharge.

### Risk factors for death from organ failure after resolution of malperfusion

**Figure 3:**
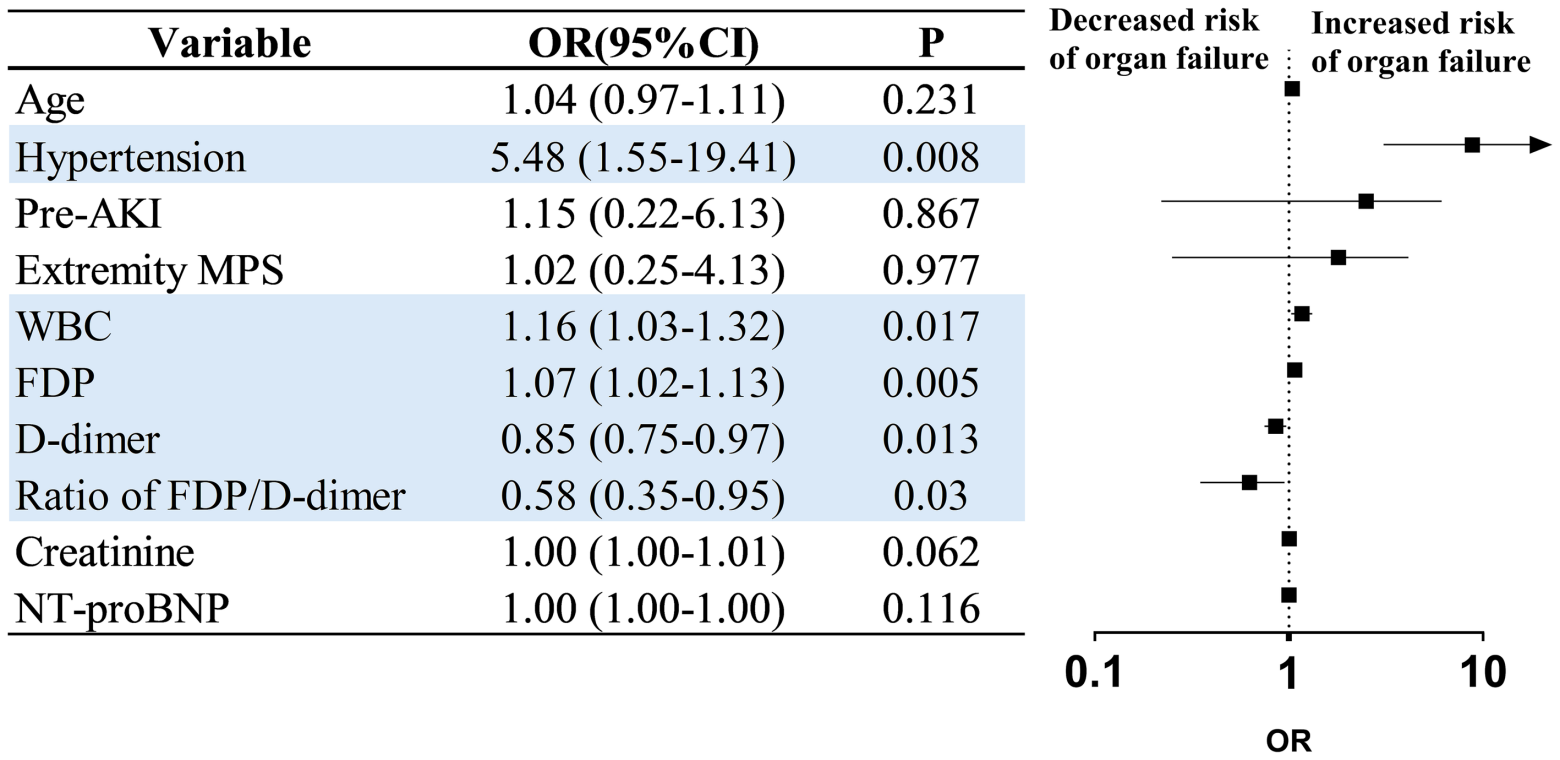
Stepwise multiple variable logistic regression illustrating associations between organ failure mortality and survival to open repair/discharge in ATAAD patients with malperfusion syndrome. Pre-AKI, preoperative acute kidney injury; WBC, white blood cell; FDP, fibrin degradation product; NT-proBNP, N-terminal pro-b-type natriuretic peptide.

Compared with patients who survived to OR or were discharged without OR, non-survivors of organ failure were older (52 vs. 47.5 years, *p* = 0.043), had higher rates of chronic kidney disease (7.1% vs. 0%, *p* = 0.013), preoperative acute kidney injury (46.4% vs. 2.4%, *p <* 0.001), and a lower prevalence of hypertension (42.9% vs. 62.4%, *p* = 0.001) (Table 3). Laboratory findings further distinguished the groups, with non-survivors exhibiting higher white blood cell (WBC) (17.1 × 10^9^/L vs. 14.1 × 10^9^/L, *p* = 0.031), fibrin degradation products (FDP) (104.5 vs. 36.1 μg/mL, *p* < 0.001), D-dimer (33.3 vs. 9.2 μg/mL, *p* < 0.001), lactate (3.7 vs. 2.7 mmol/L, *p* = 0.013), creatinine (115.5 vs. 89 μmol/L, *p* < 0.001), myoglobin levels (668 vs. 152 ng/mL, *p* = 0.023), and a lower ratio of FDP/D-dimer (3.2 vs. 3.6, *p* = 0.040) (Table 3). Univariate and multivariate analyses showed that hypertension (OR = 5.48, 95% CI 1.55–19.41), WBC (OR = 1.16, 95% CI 1.03–1.32), FDP (OR = 1.07, 95% CI 1.02–1.13), D-dimer (OR = 0.85, 95% CI 0.75–0.97), and FDP/D-dimer ratio (OR = 0.58, 95% CI 0.35–0.95) were independent risk factors for death from organ failure (Figure 3; Supplementary Table 1).

### Comparison of surgical outcomes between patients in the MPS group and those in the non-MPS group

**Figure 4:**
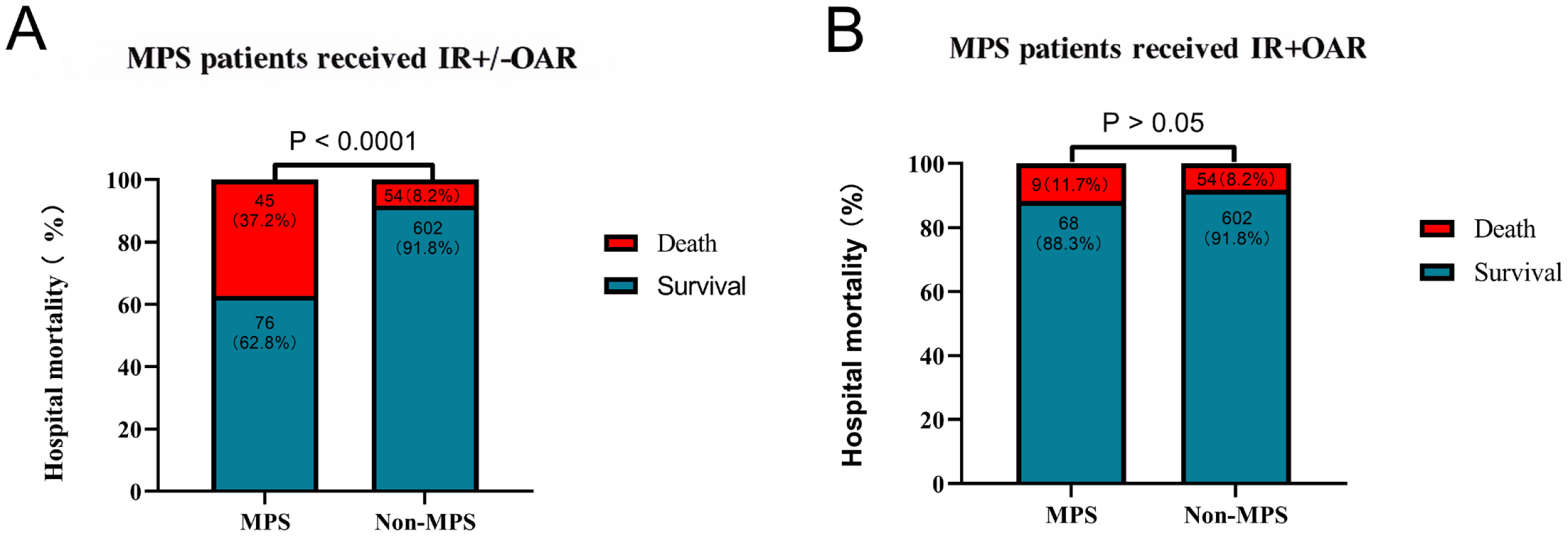
Comparison of the hospital mortality in different groups. **(A)** Overall hospital mortality (MPS vs. non-MPS); **(B)** Mortality in patients who underwent endovascular reperfusion followed by delayed repair. IR, Interventional radiology; OR, open aortic repair.

Overall in-hospital mortality was as high as 37.2% (45/121, Figure 4A), which increased with the number of organ systems involved (Supplementary Table 2). A subgroup analysis of patients who survived to delayed repair (*n* = 77, 63.6%) revealed comparable mortality to non-MPS patients (11.7% vs. 8.2%, *p* = 0.306) (Table 4; Figure 4B), supporting the fact that the strategy itself does not exacerbate organ failure when implemented in appropriately selected cases. However, the proportion of MPS patients with postoperative renal failure requiring dialysis was significantly higher (27.6% vs. 12.2% *p* < 0.001), and the postoperative length of stay (LOS) was longer (16 vs. 13 days, *p* = 0.043) (Table 4).

**Table 4:** Postoperative outcomes of patients with or without MPS.

Variables	MPS (*n* = 121)	Non-MPS (*n* = 656)	*p*-value	MPS with OR (*n* = 77)	Non-MPS (*n* = 656)	*p*-value
Reoperation for bleeding	0 (0)	9 (1.4)	0.188	0 (0)	9 (1.4)	0.305
New-onset CVA	7 (5.7)	25 (3.8)	0.335	6 (7.8)	25 (3.8)	0.101
Tracheostomy	5 (4.1)	27 (4.1)	0.979	5 (6.6)	27 (4.1)	0.334
Requiring new dialysis for acute kidney failure	21 (17.1)	80 (12.2)	0.140	21 (27.6)	80 (12.2)	* < 0.001
Chest infection	2 (1.6)	8 (1.2)	0.969	2 (2.6)	8 (1.2)	0.324
Total LOS (days)	11 (2, 17)	13 (11, 20)	0.544	16 (13, 20)	13 (11, 20)	0.043
In-hospital mortality	45 (37.2)	54 (8.2)	* < 0.001	9 (11.7)	54 (8.2)	0.306

MPS, malperfusion syndrome; Non-MPS, no malperfusion syndrome; OR, open aortic repair; CVA, cerebrovascular accident; LOS, length of stay. *p < 0.05 was considered to be significantly different.

### Short-term survival

**Figure 5:**
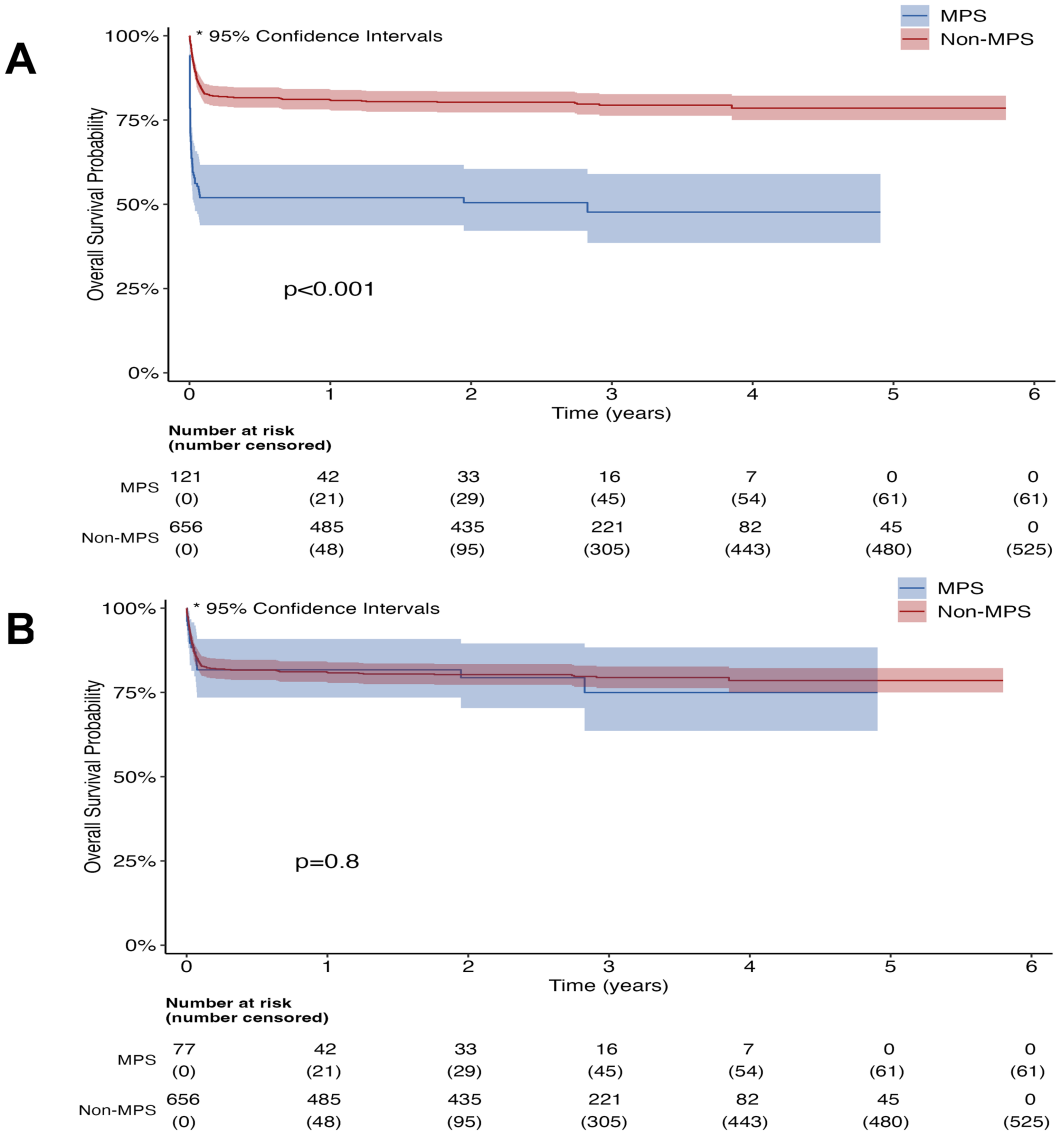
Five-year survival in patients with malperfusion syndrome (MPS) versus non-MPS. **(A)** Survival since hospital admission, all MPS patients (*n* = 121) versus non-MPS patients. **(B)** Survival since open repair, surgical MPS patients (*n* = 77) who underwent open repair versus non-MPS patients.

The median follow-up time was 1.7 years. Overall short-term survival was poorer in patients with MPS group (Figure 5A); however, among patients who survived and underwent central repair, short-term survival was similar to that of patients without MPS (Figure 5B). In addition, short-term stent patency was successfully achieved, and no severe complications, such as distal arterial embolism or spinal cord ischemia, occurred in any of the stent grafts performed at our hospital.

## Discussion

The main findings of this study includes the following: (1) overall in-hospital mortality was significantly higher in ATAAD patients with MPS than that in ATAAD patients without MPS (37.2% vs. 8.2%, *p* < 0.001); (2) the subgroup of MPS patients who successfully underwent delayed open aortic repair following endovascular therapy demonstrated comparable hospital mortality rates (11.7% vs. 8.2%; *p* = 0.306) and short-term survival outcomes comparable to non-MPS patients; (3) a multivariate analysis identified hypertension, leukocytosis, FDP, and D-dimer levels as independent predictors of organ failure-related mortality; and (4) aortic and/or branch vessel stenting, when combined with single antiplatelet therapy, proved to be both a safe and effective reperfusion strategy with satisfactory short-term patency rates.

Endovascular reperfusion has been beneficial for malperfused patients, significantly reducing in-hospital mortality, as shown in previous studies (7, 12, 13). Notably, MPS patients who survived initial stenting and subsequently underwent delayed repair achieved comparable in-hospital mortality (11.7% vs. 8.2% mortality, *p* = 0.306) and short-term survival to non-MPS patients, indicating that the strategy does not exacerbate risk when applied to appropriately selected cases. These findings are further supported by retrospective studies affirming the safety of delayed repair after reperfusion (13, 14).

While delayed repair offers clinical benefits, the primary concern of delayed repair-aortic rupture must be weighed against the increased risk of organ failure (10). However, historical data report aortic rupture rates of 8–10% with deferred intervention in patients with ATAAD and MPS, which is not as high as would be expected (14, 15). Furthermore, the risk of organ failure is 6.6 times greater than aortic rupture in MPS populations, and shortening the interval from IR to OR to within 2 days significantly reduces both in-hospital mortality (21% vs. 11%, *p* < 0.001) and aortic rupture rates (16% vs. 4%, *p* = 0.05) (10). Accordingly, this finding is exemplified in our cohort, where preoperative mortality was predominantly driven by organ failure (23%, 28/121) rather than aortic rupture (7%, 8/121), with the median duration from IR to OR being only 0.6 days (IQR 0.4–0.9). However, due to significant geographical barriers inherent to China’s northwestern healthcare infrastructure, the symptom-to-admission interval is often more than 7 h (0.3 days, IQR 0.2–0.5), accompanied by severe organ ischemia, resulting in frequent presentations with established organ damage that necessitated salvage endovascular therapy as a bridge to definitive delayed repair. For such cases, immediate central repair may exacerbate organ malperfusion, whereas initial endovascular reperfusion facilitates organ salvage. This staged strategy aligns with emerging evidence supporting delayed definitive repair in hemodynamically stable patients with advanced organ compromise (9, 16).

Mortality attributable to end-organ MPS exceeds that of aortic rupture per se, and it is corroborated by data from the German Registry for Acute Aortic Dissection Type A (GERAADA), which identified a significant association between the affected vascular territories and 30-day mortality (17). Clinical prioritization of MPS management follows distinct anatomical considerations: (1) Extremity and mesenteric MPS were the most common presentations, and these cases were associated with severe comorbidities, such as hypotensive shock and metabolic acidosis, necessiating immediate revascularization to prevent irreversible tissue necrosis; (2) In cerebral MPS, 12 cases underwent extra-anatomic revascularization before aortic repair and achieved favorable neurological recovery. The remaining two patients presented with coma and profound neurological deficits; technical success was achieved in both, but one patient succumbed to multi-organ failure. (3) One hemodynamically stable patient with coronary MPS (evidenced by ECG changes and troponin elevation) underwent immediate coronary stenting as a bridging procedure to secure myocardial perfusion before being transferred for emergent open aortic repair. In this case, the coronary intervention was deemed necessary to mitigate the high risk of perioperative cardiac catastrophe during the definitive aortic procedure. (4) Renal MPS poses unique challenges, typically co-occurring with mesenteric or extremity malperfusion. Despite this finding, renal artery stenting was selectively employed (5/32), reflecting our center’s therapeutic hierarchy prioritizing mesenteric/limb salvage over renal revascularization. This strategy is grounded in the renal system’s relative tolerance of transient ischemia during cardiopulmonary bypass, owing to its dual arterial supply and adaptive autoregulatory mechanisms.

Conversely, Brown et al. (18) have advocated for a strategy of central repair since only 7.4% of patients with MPS required reinterventions, and the hospital mortality was acceptable. Notably, reports have shown that immediate central repair within 5 or 6 h of symptom onset has a low rate of postoperative mortality and favorable cumulative 5-year survival (19, 20). Whether surgical treatment is immediate or delayed, the time of symptom onset to operation emerges as a critical determinant of outcomes in MPS management. Chen et al. (20) reported that delayed open surgery in late-presenting patients (>6 h) reduced mortality by 60% versus immediate surgery (25% vs. 62.5%). In our center, given the prolonged symptom onset (7 h) and severe end-organ compromise, open aortic repair carried prohibitively high operative risks. Therefore, endovascular intervention served as a salvage strategy to mitigate life-threatening ischemia while avoiding futile radical procedures in this critically ill cohort.

Beyond debates over procedural timing, biomarkers such as FDP and D-dimer play a critical role in risk stratification and treatment selection in patients with MPS. Aortic dissection involving the terminal branches and endothelial injury activated both the coagulation and fibrinolysis systems, and the clinical manifestations can range as a spectrum of either bleeding or thrombotic manifestations (21, 22). D-dimer, a stable and smallest end-product of fibrin degradation, reflects the dynamic equilibrium between fibrin formation and dissolution (23, 24), while FDP and D-dimer demonstrate high diagnostic and prognostic utility in aortic dissection (25–28). Elevated D-dimer (>8.3 μg/mL) correlates with extensive dissection and higher mortality (29), a finding corroborated by our analysis showing significantly elevated FDP (104.5 vs. 36.1 μg/mL, *p* < 0.001) and D-dimer (33.3 vs. 9.43 μg/mL, *p* < 0.001) in non-survivors of organ failure, indicative of severe ischemia and widespread vascular compromise. Additionally, elevated levels of FDP and D-dimer demonstrate significant interaction with inflammatory response systems, primarily through the induction of leukocyte adhesion and recruitment, resulting in damage to the vascular endothelium (25). In this study, we specifically identified elevated leucocytes that act as an independent risk factor for organ failure progression. Notably, when emergency open aortic repair is performed, the concomitant inflammatory response and cardiopulmonary bypass may synergistically exacerbate coagulation cascade activation, potentially accelerating the pathophysiological process (21, 25, 30).

Critically, the elevation of FDP and D-dimer may serve to guide recanalization therapy strategies. Two extremity MPS cases (onset >0.6 days) presented with profoundly elevated D-dimer and FDP levels, and complete thrombosis. Both required adjunctive thrombolysis following revascularization through stenting due to persistent obstruction and an unrevascularized lumen. Despite compartment syndrome (fasciotomy) and hypermyoglobinemia (dialysis), neither patient required amputation or succumbed to fatal organ failure, suggesting aggressive intervention improves outcomes in advanced MPS. While systemic thrombolysis remains controversial in ATAAD due to rupture risks, targeted endovascular thrombolysis or thrombectomy may benefit select MPS patients (10). Under the guidance of D-dimer, endovascular thrombolysis with low-dose rtPA is safe and effective (31). This finding aligns with studies showing D-dimer elevation after coronary recanalization and cerebral reperfusion (32–34), supporting its role in monitoring reperfusion efficacy. Collectively, we advocate for biomarker-driven strategies-combining blood pressure control, anti-inflammatory measures, and judicious thrombolysis to mitigate organ failure in advanced MPS cases.

Our data suggest that the endovascular-first strategy is viable primarily for patients who respond to initial reperfusion and can be bridged to surgery, rather than being a universally successful approach for all stable MPS patients. During follow-up with single antiplatelet therapy, short-term stent patency was achieved, and no severe complications occurred in any of the stent graft cases at our hospital, such as distal arterial embolism or spinal cord ischemia. Therefore, considering the simple and minimally invasive stent implantation procedure, stenting of the aorta and/or branches appears to be the preferred reperfusion strategy.

Our study, however, has certain limitations. This was a retrospective study with a relatively short follow-up period and a small sample size of patients with ATAAD with MPS. The absence of a direct internal control group for immediate repair limits definitive claims of superiority for the delayed strategy. Nevertheless, the results should be interpreted as evidence of the feasibility and outcomes of a specific protocol in a selected patient population, thereby contributing to the collective body of evidence alongside other reported strategies. Limited to a single-center institution and with a prolonged duration of symptom onset to admission, we propose possible strategies for patients with MPS and ATAAD. Multi-institutional registry studies with more patients are required in the future.

## Conclusion

For ATAAD patients with MPS, endovascular reperfusion followed by delayed open repair achieved comparable in-hospital mortality and short-term survival outcomes to those of non-MPS patients. With 23% organ failure mortality, late presentation (>7 h) rather than surgical delay is the primary factor. Biomarkers such as hypertension, leukocytosis, FDP, and D-dimer levels effectively identified high-risk patients and may inform adjunctive thrombolysis. Aortic and branch stenting serves as a minimally invasive bridging strategy with satisfactory short-term patency. Collectively, these findings support an endovascular-first approach for MPS, although further multi-institutional studies are needed to optimize patient selection and refine treatment timing.

## Data Availability

The original contributions presented in the study are included in the article/Supplementary material, further inquiries can be directed to the corresponding author.

## Author’s note

This manuscript was presented at the AATS Annual Meeting at the Metro Toronto Convention Center, Toronto, Canada, on 4/28/2024.

## Supplementary material

SUPPLEMENTARY VIDEO 1 Management of ATAAD patients with malperfusion syndrome.
