# Supplementary material for: Endovascular reperfusion followed by delayed open aortic repair in stable acute type A aortic dissection with malperfusion syndrome: a single-center experience
Source: Front Med (Lausanne). 2026 Feb 11;12:1701176. doi: 10.3389/fmed.2025.1701176 (PMC12933946; doi:10.3389/fmed.2025.1701176)
Supplement: Supplementary file 1 [file Table_1.docx]

**Extra-anatomic revascularization for cerebral MPS**

Our institutional surgical strategy for addressing ATAAD in the presence of cerebral malperfusion has been previously published (PMID: 37539273). Briefly, extra-anatomic revascularization and a new cannulation strategy were applied in patients with cerebral MPS with occluded aortic branch vessels. All procedures were performed via median sternotomy. After femoral artery cannulation, the occluded innominate artery, right common carotid artery, or left common carotid artery was fully exposed above the normal site. Thrombus from the dissected vessels was removed, and a prosthetic graft was anastomosed proximal to the occlusion site. The distal end of the prosthetic graft was then anastomosed to the extracorporeal circulation pump tube, enabling early selective cerebral perfusion via the prosthetic graft.

Supplementary table 1. Univariate logistic regression analysis for factors contributing to ATAAD patient with malperfusion syndrome (MPS) presenting with death associated with organ failure

| Variables | OR (95%CI) | P |
| --- | --- | --- |
| Age | 1.05 (1.00-1.09) | ^*^0.043 |
| Gender, male | 0.73 (0.19-2.80) | 0.646 |
| BMI | 0.99 (0.96-1.02) | 0.608 |
| Hypertension | 2.21 (0.93-5.26) | 0.073 |
| Extremity MPS | 2.71 (1.13-6.51) | ^*^0.025 |
| Mesenteric MPS | 0.52 (0.21-1.31) | 0.164 |
| Pre-AKI | 0.27 (0.11-0.67) | ^*^0.005 |
| WBC | 1.10 (1.01-1.20) | ^*^0.024 |
| FDP | 1.01 (1.01-1.02) | ^*^<0.001 |
| D-dimer | 1.04 (1.02-1.06) | ^*^<0.001 |
| Ratio of FDP/D-dimer | 0.71 (0.49-1.02) | 0.064 |
| Creatine | 1.00 (1.00-1.01) | ^*^0.019 |
| Myoglobin | 1.00 (1.00-1.00) | 0.730 |
| NT-BNP | 1.00 (1.00-1.00) | ^*^0.012 |

BMI, body mass index; Pre-AKI, preoperative acute kidney injury; FDP, fibrin degradation product; WBC, white blood cell; FDP, fibrin degradation product; NT-proBNP, N-terminal pro b-type natriuretic peptide. *, p<0.05.

Supplementary table 2.Survival per Number of Pre-Operative Malperfused Organ Systems

| Malperfused organ systems | Total | Survivors | Dead | Percent dead per group |
| --- | --- | --- | --- | --- |
| None | 656(84,4) | 602(77.4) | 54(6.9) | 8.2 |
| 1 | 19(2.4) | 14(1.8) | 5(0.6) | 4.1 |
| 2 | 47(6.0) | 33(4.2) | 14(1.8) | 11.6 |
| ≥3 | 55(7.1) | 29(3.7) | 26(3.3) | 21.5 |
